# Microplastic Removal from Drinking Water Using Point-of-Use Devices

**DOI:** 10.3390/polym15061331

**Published:** 2023-03-07

**Authors:** Ashlyn G. Cherian, Zeyuan Liu, Michael J. McKie, Husein Almuhtaram, Robert C. Andrews

**Affiliations:** Department of Civil and Mineral Engineering, University of Toronto, Toronto, ON M5S 1A4, Canada

**Keywords:** membranes, microfiltration, granular activated carbon, ion exchange, polyethylene terephthalate, polyvinyl chloride, nylon

## Abstract

The occurrence of microplastics in drinking water has drawn increasing attention due to their ubiquity and unresolved implications regarding human health. Despite achieving high reduction efficiencies (70 to >90%) at conventional drinking water treatment plants (DWTPs), microplastics remain. Since human consumption represents a small portion of typical household water use, point-of-use (POU) water treatment devices may provide the additional removal of microplastics (MPs) prior to consumption. The primary objective of this study was to evaluate the performance of commonly used pour-through POU devices, including those that utilize combinations of granular activated carbon (GAC), ion exchange (IX), and microfiltration (MF), with respect to MP removal. Treated drinking water was spiked with polyethylene terephthalate (PET) and polyvinyl chloride (PVC) fragments, along with nylon fibers representing a range of particle sizes (30–1000 µm) at concentrations of 36–64 particles/L. Samples were collected from each POU device following 25, 50, 75, 100 and 125% increases in the manufacturer’s rated treatment capacity, and subsequently analyzed via microscopy to determine their removal efficiency. Two POU devices that incorporate MF technologies exhibited 78–86% and 94–100% removal values for PVC and PET fragments, respectively, whereas one device that only incorporates GAC and IX resulted in a greater number of particles in its effluent when compared to the influent. When comparing the two devices that incorporate membranes, the device with the smaller nominal pore size (0.2 µm vs. ≥1 µm) exhibited the best performance. These findings suggest that POU devices that incorporate physical treatment barriers, including membrane filtration, may be optimal for MP removal (if desired) from drinking water.

## 1. Introduction

Microplastics (MPs) have been reported in aquatic environments worldwide, including both marine and freshwater environments [1,2] which serve as the predominant sources of drinking water for human consumption. Microplastic concentrations in these source waters are anticipated to increase over time [3]. Limited prior studies have confirmed the presence of MPs in untreated water that is used by drinking water treatment plants (DWTPs) [4,5,6], with MP concentrations reaching up to >4000 particles per liter. Although conventional DWTPs have been reported to remove 70 to >90% of MPs ≥ 1 μm [7,8], treated drinking water may represent a potential source of MPs for consumers, given that billions of liters are produced daily at facilities around the globe [4,5,9,10]. In addition, the drinking water supply chain itself may serve as a potential source of microplastic contamination in drinking water [8,9]. Previous studies have also reported the presence of microplastics in tap water [6,10,11]. Due to limitations in the technologies employed by conventional water treatment facilities and given the physical properties of MPs, challenges exist with respect to their removal [9]. 

The filtration of treated drinking water by consumers using point-of-use (POU) devices has gained acceptance and popularity in part due to their previous application for the removal of particulate lead that may originate from service lines and plumbing materials [12,13]. POU devices have been widely employed to remove heavy metals, including lead, arsenic, and copper, as well as fluoride, nitrate, and taste and odor compounds from drinking water [14]. Consumers can potentially satisfy their desire to increase the removal of undesirable substances by utilizing filters that have been tested by an accredited third-party certification body or bodies for lead and particulate reduction (Class I) capabilities, with respect to American National Standards Institute (ANSI)/National Sanitation Foundation (NSF) health-based performance standards (Standards 42 (Class I particulate, 0.5–1 µm, chlorine taste and odor, particulate matter and turbidity) and 53 (dissolved and particulate, ≤0.45–1 µm lead, chromium and mercury). However, unlike NSF-53 certified pour-through POU devices, NSF-42 certified devices are not rated for the removal of particulates <30 µm in size (highest level of NSF-52 certification observed for particle removal using pour-through POU devices is class 5, i.e., the removal of particles >30–50 µm), as particles <30 µm are likely to pass directly into filtered water [14]. Previous studies have evaluated common POU devices that achieve NSF-53 and NSF-42 certification standards for the removal of dissolved (≤0.45 µm [14,15,16,17]) and particulate matter (0.45–1 µm; [13]); however, the removal of MP particles has not been specifically addressed. 

In 2015, approximately 51% of Canadian households used some type of home filtration device to treat drinking water [18]. These devices incorporate various approaches to treat water immediately prior to use (e.g., cooking and drinking), including granular activated carbon (GAC), solid block activated carbon (SBAC), ion exchange (IX) resin, and reverse osmosis (RO). Such treatment approaches have the ability to simultaneously remove a number of contaminants from aqueous solutions, including dissolved organics and ions, inorganic particles [14,19] total suspended solids (TSS), particulate lead, and other heavy metals. Given that MPs are considered to be inorganic particles [20], these types of treatment methods should be able to remove MPs via the same mechanisms (i.e., physical interception). Activated carbon-based filters have been proven to be effective in removing inorganic particles via granular filtration or adsorption [19,21]. GAC, with a high and non-uniform porosity, typically provides a large surface area (500–1500 m^2^/g) and is effective for the removal of particulate matter [22]. To achieve the treatment of a wider range of compounds, GAC may be incorporated into POU filters, along with IX resin. IX can consist of anion exchange (AX) or cation exchange (CX) resin and the latter is commonly incorporated to preferentially exchange charged inorganic species. When considering GAC and IX, performance may decrease as adsorption and ion exchange sites are occupied by organic compounds or ionic metal species such as iron, magnesium, and copper. SBAC may consist of activated carbon particles that are fused together, resulting in a block that exhibits smaller pore sizes (0.5–1.0 µm), which are capable of intercepting inorganic particles [14]. Based on our understanding of the mechanisms associated with lead removal within the filters along with theoretical evidence, it is anticipated that POU filters certified in accordance with NSF-53 and NSF-42 standards may be effective in the removal of microplastics >1 µm. These are typically the most challenging to remove using physical processes, and as a result, particles greater than this size should be more effectively controlled. 

The primary objective of this study was to evaluate and compare the relative performance of three common POU devices that employ combinations of GAC, IX, MF, and non-woven membranes (MEM), with respect to MP removal from treated drinking water. It was hypothesized that MP physical characteristics (e.g., shape, polymer type, size, etc.), the type of treatment applied, and remaining capacity could influence their efficiency. The results of this study may provide guidance to consumers who desire to potentially remove MPs from municipally treated drinking waters. 

## 2. Materials and Methods

### 2.1. Point-of-Use Treatment Devices

Details pertaining to the three POU devices examined, including the treatment technologies incorporated, certifications, and operational guidelines, are summarized in Table 1. All devices considered were available at major retailers in North America.

POU Device 1 (GAC + IX) incorporates coconut-based GAC along with IX resin, whereas POU Device 2 (GAC + IX + MEM) is composed of a 5-stage filter (coarse filter screen, foam distributer, multi-layer GAC and oxidation reduction alloy, IX resin, along with ultra-fine screens and non-woven membrane layers (pore size ≥ 1 µm). Non-woven membranes typically consist of continuous, interlocked fibers and are highly porous and permeable [23]. POU Device 3 (MF + GAC + IX) incorporates GAC with IX resin along with a membrane microfilter (pore size ≥ 0.2 µm). All devices currently satisfy ANSI/NSF 42 and 53 protocols. It is recommended by the respective manufacturers that all devices that incorporate GAC/IX are replaced following 150 L or approximately every two months; the replacement of membrane-based systems (POU Device 3) is recommended following 1000 L or once per year. 

### 2.2. Preparation of Stock Solutions

**Figure 1:**
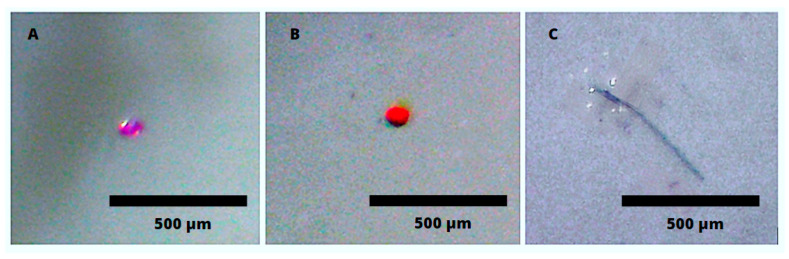
Spiked microplastics: pink PET fragment (**A**), orange PVC fragment (**B**); green nylon fiber (**C**).

To avoid the potential variability in microplastic concentration and composition, stock solutions of microplastics were spiked into municipally treated tap water. Among the MPs prevalent in drinking water, fragments and fibers are the most abundant morphotypes [4,6]. As such, a combination of varying particle shapes, sizes, and compositions was used to assess the removal of MPs (Table 2; images of representative particles are shown in Figure 1). To ensure consistency, large sample volumes (>15 L) were employed, along with consistent preparation and sampling protocols. 

To prepare stock solutions, 10 mg of clear polyvinyl chloride (PVC) and polyethylene terephthalate (PET) fragments, ranging in size from 10 to 300 µm (Custom Processing Services, Reading, PA, USA), were dyed orange and pink, respectively, using 5 mg/mL solutions of iDye Poly powder (Jacquard, Healdsburg, CA, USA), following a method described by Tewari et al. [24]. Particles were dyed to simplify the subsequent microscopic identification and enumeration, as well as to eliminate the need for characterization by FTIR or Raman spectroscopy. Particles of these colors were not identified in the tap water that was spiked. During the dyeing process, plastics were placed in a dye solution heated to 65 ± 5 °C and mixed for 1 h; all the beakers were completely covered with aluminum foil to prevent airborne contamination. After allowing the solutions to cool to room temperature (22 °C), they were poured into 15 mL polypropylene (PP) tubes (Eppendorf 5804R, Hamburg, Germany) and centrifuged at 1000 rpm for 5 min to separate microplastic particles from the dye solution. The supernatant was removed using a glass serological pipette and replaced with 10 mL of Elix^®^ water (15 MΩ∙cm) (MilliporeSigma, Oakville, ON, Canada) and 5 mL ethanol, which had been filtered through 0.45 µm pore size glass fiber filters to remove any potential contamination by airborne microplastics; the process was repeated until the supernatant was free of dye. Microplastics in solution were stored in clear glass jars until use. Green nylon fibers were prepared by cutting nylon thread with a diameter of 33 ± 2 µm into <1 mm lengths using a scalpel (Flock It, Rockford, IL, USA). 

### 2.3. Preparation of Spike Solutions

To prepare a spike solution that contained a known number of microplastic particles or fibers, 200 μL of each dyed-particle solution was pipetted onto a 47 mm diameter 10 μm pore size polycarbonate (PC) filter (Millipore Sigma, Burlington, MA, USA). Particles retained on the filter following vacuum filtration were counted using an OMAX 10×–80× binocular zoom stereo microscope (Microscopenet.com, Kitchener, ON, Canada). The microscope was calibrated using a stage micrometer (Wards Science, Rochester, NY, USA). Filters that retained a known number of plastic particles were placed in sealed glass jars containing 250 mL of RO water and sonicated in an ultrasonic bath (5 L, 155 V; Fritsch Equipment Corp., De Pere, WI, USA) for 15 min. The filter was subsequently removed, rinsed, and inspected such that any remaining particles could be subtracted from the initial count. RO water that contained a known number of particles was employed as a spiking solution. The average number of particles spiked for each specific polymer type, as well as the associated average major and minor dimensions, are shown in Table 2. 

### 2.4. Laboratory Control Measures

It is crucial to use plastic-free equipment and conduct analyses in a dust-free environment to limit procedural sample contamination [25]; hence, glassware and stainless-steel containers were used whenever possible. All the labware was dried and stored along with the samples and filtration was conducted in a lab equipped with a HEPA air filter (ALEN, Austin, TX, USA), which removes 99.99% of particles ≥0.3 µm. All the labware was thoroughly washed with non-phosphate detergent, rinsed six times with RO water, and dried in clean air prior to use. Samples were collected in 1 L glass beakers and covered with foil to reduce potential atmospheric contamination [26]. All work surfaces, particularly on and around the microscope, were wiped with a 70% ethanol solution [27,28]. To account for the possible deposition of airborne fibers from white cotton lab coats and ensure accurate quantification, laboratory blanks were analyzed in duplicate [29].

### 2.5. System Blanks

System blanks were prepared and collected in duplicate to identify any potential particle loss/addition due to sample storage and dispensing. A system blank consisted of a clean sample (e.g., 1 L sample of microplastic-free Elix water) that was collected prior to each spiked sample. The system blank was subjected to all steps involved in preparation, filtration, and extraction using the same equipment. Following extraction, the system blank was visually examined using a microscope and classified in the same manner as 1 L samples. Suspected microplastics that resembled a contaminant, possibly as a result of particle accumulation due to factors other than the treatment itself (e.g., sorption to containers), were recorded and used to ensure background levels of microplastics remained at <10% of the total reported values [27], thereby minimizing the presence of false positives. 

### 2.6. Spike and Recovery

An initial set of spike-and-recovery tests (*n* = 4) were conducted using POU Device 1 (GAC + IX; in addition to the two replicates used for subsequent trials) to determine the performance of the analytical procedure. Thorough rinsing with a surfactant solution has been shown to be important to ensure microplastic recovery [8]. As such, following filtration, 20 L stainless-steel Cornelius Ball Lock Kegs (Ontario Beer Kegs, Bolton, ON, Canada), which were used to ensure a representative quantity of particles [30], were triple rinsed using 1% solution of Alcojet detergent, a non-ionic surfactant (Alconox, White Plains, NY, USA). The Alcojet solution was prepared by heating Elix water to 60 °C, stirring the powdered detergent until it was dissolved, then passing it through a 10 μm PC filter. The rinsed water was subsequently passed through a 10 μm PC filter. Similar to other methods for other analytes, recovery values of ≥80% were required [31]. Recovery may potentially be reduced by particle accumulation due to factors other than the treatment itself (e.g., sorption to containers). Method recovery values were calculated. 

A mean recovery efficiency of 94% was achieved for an initial set of spike-and-recovery trials with POU Device 1 (GAC + IX). Subsequent spike-and-recovery tests were conducted following the collection of each 1 L spiked sample (approximately following every 25% increase in POU capacity). Any spiked particles that remained following the rinsing of the sample storage containers were subtracted from the total number of particles initially dosed in an effort to help to ensure that the removal of particles was truly reflective of the particles dosed into the POU device. 

### 2.7. Spiking Microplastics into POU Devices

Concentrations of 39 ± 9 PVC, 36 ± 7 PET, and 64 ± 15 nylon fibers/L were used in this study. In addition, 15 L of Elix water was spiked with a known number of microplastics to achieve a concentration of 139 ± 31 particles/L among all the microplastic types in a closed stainless-steel keg that was used to supply sample water. Each POU filter was initially flushed with 2 L of Elix water, as recommended by the manufacturer, to remove any carbon particles. A 1 L sample of Elix water was filtered, collected, and analyzed to serve as a “device blank”, so that any background contamination could be identified. Microplastic-spiked Elix water was then transferred under pressure (to 145 kPa, or approximately 20 psi) from the keg using an air compressor (Mastercraft, Mississauga, ON, Canada). The water was directly discharged via 6.35 mm (¼ inch) stainless-steel tubing to each POU device. Subsequent 1 L samples were analyzed following 25%, 50%, 75%, 100%, and 125% of the rated treatment capacities. To ensure that rapid deterioration of the treatment performance did not occur at 100% of the rated capacity, additional samples were collected and analyzed up to 125% of the rated treatment capacity. 

### 2.8. Quantification of Microplastics

First, 1 L samples were collected following each 25% increment in the rated capacity. Each supply container was triple rinsed using a 1% solution of Alcojet detergent, which was collected in 1 L glass beakers. The rinsed water was subsequently passed through a 10 μm PC filter, which was placed in a clean glass petri dish (VWR, Mississauga, ON, Canada).

A transparent 0.5 cm grid (Diversified Biotech, Dedham, MA, USA) was affixed to the bottom of the petri dishes prior to particle counting, using an OMAX 10×–80× binocular zoom stereo microscope (Microscopenet.com, Kitchener, ON, Canada). One grid was counted at a time, row by row, right to left. Each microplastic particle was classified by shape and color, using forceps to prod if needed. Differences in the mean recoveries between two POU devices of the same type were examined using a Student’s *t*-test. Statistical analyses were performed using R 3.6.2.

### 2.9. Mass Balance around Microplastic Analysis

Plastic particles that entered the POU devices included those that were spiked into the 15 L sample supply (keg). Influent microplastics were enumerated using microscopy, such that any potential losses associated with plastic particles that settled to the bottom of the keg or adhered to the discharge line or the POU device itself were considered by subtracting any remaining particles from the initial count. Removal was attributed to the remaining plastic particles. 

## 3. Results and Discussion

### 3.1. Blanks

The number of suspected microplastics in the system blank samples was used to assess any potential variability that was not related to the POU devices and to help to ensure that the background microplastic levels were <10% of the total reported values in an effort to eliminate false positives [27]. On average, system blanks contained 0–3 microplastics (when considering all three devices), thereby validifying the absence of false positives. 

### 3.2. Microplastic Recovery Determination

**Figure 2:**
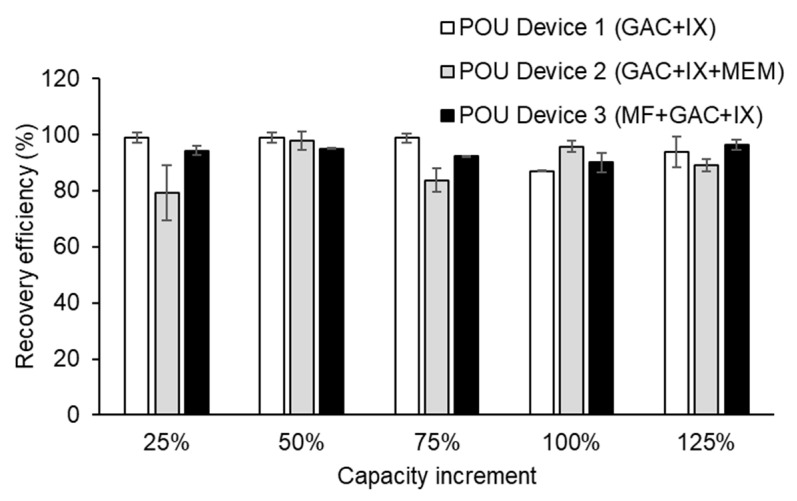
Mean particle recovery following each 1 L sample filtered through duplicates of POU Device 1 (GAC + IX), POU Device 2 (GAC + IX + MEM), and POU Device 3 (MF + GAC + IX). Vertical bars represent ± one standard deviation (*n* = 2).

A mean recovery of 94.3 ± 2.9% was observed when considering particles that varied in polymer type, size, and shape during the initial spike and recovery trials for POU Device 1 (GAC + IX). Additional spike and recovery calculations were performed following every 25% increase in the POU capacity for all three devices (Figure 2). A mass balance was conducted for all samples to determine the true removal value associated with each POU device. Similar to POU Device 1, high recoveries were observed for POU Devices 2 (GAC + IX + MEM) and 3 (MF + GAC + IX), with replicates demonstrating mean recovery efficiencies of 90 ± 5.9% and 93.6 ± 2.2%, respectively. These data confirm that any potential particle loss or accumulation associated with the sampling process did not impact the initial concentrations dosed into the POU devices. 

### 3.3. MP Removal by POU Devices

**Figure 3:**
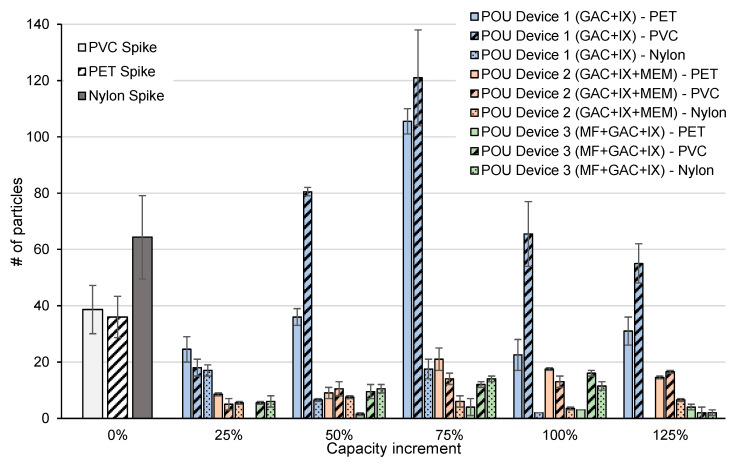
Mean microplastic particles or fibers in POU effluent as a function of rated treatment capacity. Vertical bars represent ± one standard deviation (*n* = 2).

The mean effluent MP particles for each of the three polymer types and POU devices as a function of rated treatment capacity are shown in Figure 3. POU Device 1 (GAC + IX) exhibited inferior performance with respect to the removal of PET and PVC fragments, where PET particles in the effluent exceeded the influent at 75% of the rated capacity and PVC at 50–125%. The observed increase may potentially be attributed to the accumulation and release of trapped particles. In contrast, the nearly complete removal of nylon fibers was achieved when the capacity reached 100% for POU Device 1 (GAC + IX). These results suggest that the POU devices that only incorporate GAC and IX may not effectively remove microplastics, despite meeting ANSI and NSF health-based performance certifications. GAC and IX are typically employed for the chemical adsorption of organic and ionic contaminants and not the physical removal of particulates [32,33]. 

**Figure 4:**
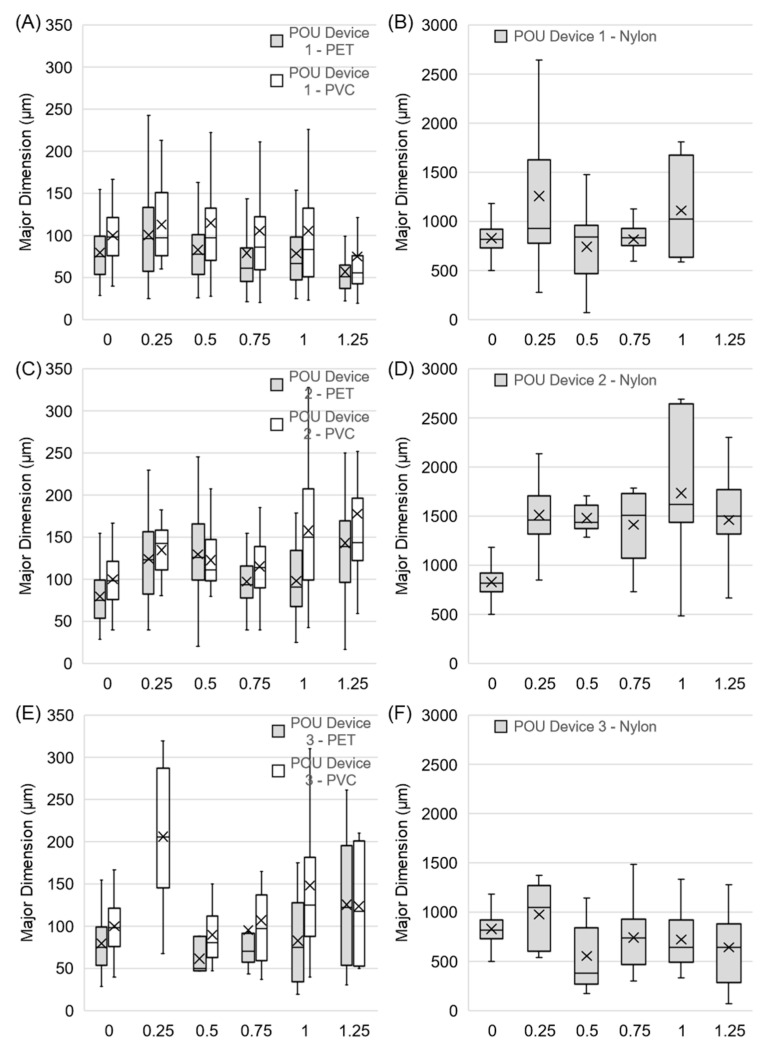
Change in size distribution of the major dimensions of PET, PVC, and nylon microplastics as a function of the increasing rated treatment capacity for all three POU devices. X represents the mean and lines represent the maximum, 3rd quartile, median, 1st quartile, and minimum values, from top to bottom.

POU 2 (GAC + IX + MEM) and 3 (MF + GAC + IX) that incorporated membrane filtration exhibited considerably better performance, especially for the removal of PVC and PET fragments, which ranged from 46 to 78% and 54 to 86%, respectively, for POU 2, while those of POU 3 ranged from 90 to 100% and 55 to 94%, respectively. POU 3 (MF + GAC + IX) exhibited the best overall performance for fragment removal, but was comparable to POU 2 (GAC + IX + MEM) when considering fiber reduction. However, fragment removal decreased with increasing capacity increments for both POU 2 and 3. No significant loss of performance (level of significance α = 0.05) was observed for any of the POU devices at 125% capacity. In addition, the preferential removal of fragments and fibers of any specific size was not observed (Figure 4). Greater overall removal by POU 3 could be attributed to the smaller membrane pore size of 0.2 µm when compared to that of POU 2 (≥ 1 µm), which was claimed by the manufacturer to remove 99.999% of all MPs from drinking water. Higher removals by POUs 2 and 3 can be attributed to the incorporation of membrane filters, which are known to remove microplastics [34]. Despite the small pore sizes associated with the membrane-based filters, microplastics were observed in their effluents. Similar findings have been reported at full-scale drinking water treatment facilities, where microplastics have been reported in the effluent of ultrafiltration and reverse osmosis filters [35,36]. It could be hypothesized that this could potentially be due to imperfections in the membrane surfaces, resulting in pores larger than the nominal size.

Greater removal of PET fragments (100 ± 33 µm) was observed when compared to smaller PVC fragments (79 ± 32 µm) for POUs 1 and 3, while the removal of both polymer types was similar for POU 2 for all increments with the same capacity (Figure 3). These data suggest that in general, POU devices preferentially remove larger fragments, as anticipated based on previous literature [14]. In addition to particle size, removal is impacted by material shape. Greater removal of nylon fibers (826 ± 157 µm in length, 33 ± 2 µm in diameter) was observed when compared to PVC and PET fragments for all POUs. Fibers may represent the most common microplastic morphology in drinking waters, depending on the source water. Studies that examined drinking water treatment facilities drawing from rivers report a greater abundance of fibers when compared to fragments or spheres [4,6,8,37], whereas groundwater sources typically contain few fibers [5,38]. The removal of fibers in conventional drinking water treatment has been associated with sedimentation and filtration processes; however, this reduction is typically limited to 70–80% [6,8,37]. Fibers are especially associated with breakthroughs during treatment when compared to fragments or spheres [39]. As a result, microplastic fibers may be observed in treated drinking waters; Cherniak et al. reported a concentration of 19 fibers/L in a full-scale drinking water distribution system [8]. It is suggested that future studies should specifically examine the removal of small particles (1–20 µm), which have been reported in drinking water [4] and may have implications regarding human health [40,41,42,43,44]. Further investigation is also required to evaluate a wider range of POU device treatment technologies that would include solid block activated carbon and reverse osmosis and other types (faucet and under-the sink) with a larger sample size.

## 4. Conclusions

This is the first known study to evaluate three readily available and easy-to-use POU devices for MP removal. The results suggest that 95–100% removal of 79 ± 32 µm PVC and 100 ± 33 µm PET fragments, as well as 826 ± 157 µm by 33 ± 2 µm nylon fibers, can be achieved. MP removals were highest for the POU device with the smallest pore size membrane filters, while the device that only incorporated GAC and IX exhibited poor performance, including effluent MP concentrations exceeding those in the influent under certain conditions. POU devices that incorporate physical treatment barriers including membrane filtration are suitable for MP removal from drinking water. Since POU devices cannot be backwashed, the media life of these devices may decrease. To ensure their effectiveness and safety, POU filters should always be operated, maintained, and replaced in accordance with manufacturer recommendations. 

## Figures and Tables

**Table 1:** Characteristics of selected POU devices.

Treatment Technologies	No. of Individual Units Tested	Estimated Maximum Capacity	Certifications	Recommended Operating Limits
POU 1 (GAC + IX)	2	150 L (GAC/IX) and 1000 L (MF)	NSF/ANSI Standards 42 and 53	• Water temperature: 0 °C–29 °C (POU 1), 2 °C–30 °C (POU 2), 0 °C–60 °C (POU 3) • Recommended replacement interval: two months (GAC/IX), one year (MF)
POU 2 (GAC + IX + MEM)	2			
POU 3 (MF + GAC + IX)	2			

**Table 2:** Characteristics and concentrations of spiked microplastics.

Composition	Color	Shape	Average Major Dimension (µm)	Average Minor Dimension (µm)	Major Dimension Size Range (µm)	Density (g/cm^3^)	Number of Particles Spiked	Source
PVC	Orange (dyed)	Fragments	79 ± 32	58 ± 27	39–246	1.1–1.35	39 ± 9	SABIC Innovative Plastics (Mt Vernon, IN, USA) ^a^
PET	Pink (dyed)	Fragments	100 ± 33	74 ± 25	28–121	1.37–1.46	36 ± 7	
Nylon	Green	Fibers	826 ± 157	33 ± 2	496–1862	1.15	64 ± 15	Flock It (Rockford, IL, USA)

^a^ Micronized by Custom Processing Services (Reading, PA, USA) and dyed as described in the work of Karakolis et al. (2019).

## Data Availability

Data are available upon request.
